# Expanding Monitoring Capacity for Potential Invasive Species in Arctic Canada With Environmental DNA Metabarcoding

**DOI:** 10.1111/gcb.70452

**Published:** 2025-09-08

**Authors:** Elizabeth Boyse, Melody S. Clark, Ian M. Carr, Alison J. Cook, Philippe Archambault, Jean E. Holloway, Zhewen Luo, Michael Milton, Mathieu Roy, Jackie Dawson, Victoria L. Peck

**Affiliations:** ^1^ British Antarctic Survey Cambridge UK; ^2^ School of Medicine University of Leeds Leeds UK; ^3^ Scottish Association for Marine Science Oban UK; ^4^ Takuvik, ArcticNet, Québec Océan Université Laval Quebec City Quebec Canada; ^5^ Department of Geography, Environment, and Geomatics University of Ottawa Ottawa Ontario Canada; ^6^ Ikaarvik Nunavut Canada

**Keywords:** Arctic, climate change, environmental DNA, human impacts, invasive species, metabarcoding, monitoring, shipping traffic

## Abstract

To date, environmental conditions have been enough to act as an effective barrier to prevent non‐indigenous species from arriving and establishing in Arctic Canada. However, rapidly changing climatic conditions are creating more suitable habitats for non‐indigenous species to potentially establish and become invasive. Concurrently, shipping traffic in parts of Arctic Canada has increased by over 250% since 1990, providing an effective vector for transporting non‐indigenous species to the region. Arctic Canada has been historically undersampled, so Arctic biota inventories are incomplete, hampering efforts to establish if a species is new to the region (and potentially invasive) or newly discovered. In this study, we utilize environmental DNA (eDNA) metabarcoding and ships of opportunity to assess eukaryotic community composition and potential invasives along one of the busiest shipping routes, the Northwest Passage. One liter seawater samples were collected in triplicate at 27 locations, targeting touristic hotspots frequently visited by passenger vessels. Eukaryotic DNA was amplified from the 18S rRNA V9 and COI regions, resulting in 126 unique Amplicon Sequence Variants (ASVs) detected with COI and 391 ASVs with 18S, providing an important snapshot of current community composition. Copepods, dinoflagellates, and diatoms were the most abundant taxonomic groups, correlating well with previous net sampler surveys, validating the efficacy of eDNA for biodiversity surveillance. We also report the first detections of a prolific invasive species, the bay barnacle (*Amphibalanus improvisus*), in Arctic Canada. Further work is currently in progress to establish whether these detections represent transient barnacle larvae or sessile adults capable of recruiting and reproducing. Our study demonstrates the utility of eDNA for the detection of non‐indigenous species in a data‐poor area, which, if combined with citizen science initiatives and local communities, could provide a vital monitoring tool for the detection of new invasives in this rapidly changing area.

## Graphical abstract

Rapidly changing climatic conditions combined with increasing shipping traffic is going to accelerate the introduction and persistence of marine invasive species in Arctic Canada. To address this emerging threat, we propose seawater environmental DNA metabarcoding on board ships of opportunity (i.e., cruise ships) as a cost‐effective method for increasing the spatiotemporal scale of monitoring in this data‐poor region. In this initial study, we report the first detections of a highly prolific invasive species, the bay barnacle (*Amphibalanus improvisus*) in the water column, underscoring the urgent requirement for increased surveillance efforts.

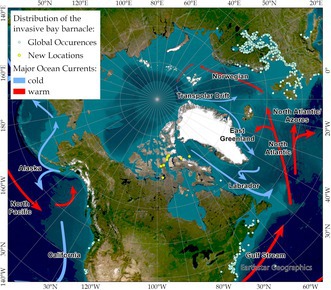

## 1 Introduction

Globally, invasive species are one of the main drivers of biodiversity loss (Anton et al. 2019; Bellard et al. 2021; Pyšek et al. 2020). Rates of invasive species introductions have intensified in recent decades as a consequence of increased globalization in synergy with other factors such as climate change (Seebens et al. 2017). The worldwide economic cost of invasive species is estimated to be over $1.28 trillion (1970–2017), of which aquatic invasions account for ~$345 billion. The value of the latter includes costs to combat damage (i.e., biofouling of infrastructure such as navigation channels, water pipes, water filters, aquaculture facilities, etc.) and management to reduce or control the spread of aquatic invaders (Cuthbert et al. 2021; Diagne et al. 2021). Due to the remoteness and extremely cold temperatures in polar regions, they have been exposed to fewer invasive species arrivals compared to other global areas. However, warming coupled with increased human activities is predicted to increase the rate of invasive species arrival to the poles (Bennett et al. 2015; Hughes et al. 2020).

The Arctic is experiencing the fastest rates of environmental change globally, with the air temperature increasing four times faster than the global average and sea ice cover declining, especially in the summer months which are predicted to become seasonally sea ice free by 2030–2050 (Jahn et al. 2024; Rantanen et al. 2022). These changing environmental conditions have resulted in larger areas of suitable habitat for high‐risk invasive species in the Arctic, and the spatial extent of these areas is expected to continue increasing (Sunday et al. 2012). At the same time, reduced sea ice has also resulted in increased accessibility of shorter, more economical maritime trade routes through the Arctic, such as the Northern Sea Route (NSR) and the Northwest Passage (NWP), connecting the Atlantic and Pacific Oceans (Mudryk et al. 2021; Ng et al. 2018). Arctic Canada has experienced a 250% increase in shipping activity since 1990, with the largest increases along the southern route of the NWP, in Frobisher Bay, and through the Hudson Strait (Dawson et al. 2018, 2022). Cargo ships make up the largest proportion of ship traffic, followed by government vessels (e.g., research vessels, icebreakers), while pleasure crafts represent the fastest growing vessel type (Dawson et al. 2018). Natural resource extraction is also contributing to shipping traffic increases, especially in Nunavut, where dry bulk vessel shipping traffic tripled between 2015 and 2019 as a direct result of the Baffinland Mary River Iron Ore Mine opening in 2015 (van Luijk et al. 2021). Increasing shipping activities are likely to result in some benefits for communities living in Inuit Nunangat (Inuit homeland in Arctic Canada) such as opportunities for more resupply ships, employment, and tourism (Olsen et al. 2019). However, increased shipping traffic also brings numerous ecological risks that could negatively impact Inuit health, culture, food security, and way of life, such as increased underwater noise, increased pollution, and the introduction of invasive species (Dawson et al. 2020; van Luijk et al. 2022).

Ships are the main vector worldwide for transporting marine non‐indigenous species to new areas, through biofouling and ballast water discharge, where they can potentially become invasive if successfully establishing a population results in negative environmental or economic impacts (Chan et al. 2016; Saebi et al. 2020; Williams et al. 2013). Only 34 marine non‐indigenous species have been reported across the Arctic's Large Marine Ecosystems (LMEs) (Chan et al. 2019; PAME 2013). This amount is comparatively low compared to elsewhere in the world; although the rate of discovery is steadily increasing (Chan et al. 2019; Vilizzi et al. 2021). Arctic LMEs at lower latitudes, including the Icelandic Shelf, the Barents Sea, and the Norwegian Sea, have experienced the highest numbers of non‐indigenous species introductions. The Northeast Atlantic has contributed to the largest number of introduction events and increased the likelihood of introduction events leading to established populations (Chan et al. 2019; Thorarinsdottir et al. 2014). In Arctic Canada (> 66°33′ N), only one marine non‐indigenous algae, *Dumontia contorta*, has been recorded (Mathieson et al. 2010). There are currently no confirmed established populations of invasive fauna, although potentially invasive benthic invertebrates have been detected, such as the tube‐dewelling amphipod, *Monocorophium insidosium*, and the tunicate *Polycarpa pomaria* (Brown et al. 2016; Goldsmit et al. 2019). A further eight species, including the Bay Barnacle *Amphibalanus improvisus*, have been identified as high‐risk invaders with potential to establish in Arctic Canada based on dispersal pathways, biological characteristics, and known invasion history elsewhere, with suitable habitat predicted in at least some regions by 2050 (Goldsmit et al. 2018). Whether introduced species persist to become invasive will then depend on a number of factors, including propagule pressure (i.e., the number of individuals released and the frequency of release events), environmental suitability, and biotic interactions with native species (Iacarella et al. 2020).

Successfully detecting non‐indigenous species in new areas relies on existing comprehensive baseline knowledge of native communities. Currently, however, the polar ocean ecosystems are the least well characterized globally as a result of being systematically under sampled. In the Arctic, this is due to challenges associated with the vast size of the coastline coupled with its remoteness (Goldsmit et al. 2014, 2019). Concerningly, Roy et al. (2015) estimated that at least 60% of benthic megafauna (species exceeding 1 cm diameter) in Arctic Canada waters remain uncharacterized (Gage and Tyler 1991; Grassle et al. 1975; Wei et al. 2020), despite the majority of potential high‐risk invaders being benthic (Goldsmit et al. 2018). Therefore, unsurprisingly, recent biodiversity studies have reported high numbers of cryptogenic species not previously recorded in Arctic Canada owing to increased survey effort and new technologies, such as environmental DNA (eDNA) surveys, although many are not suspected to be invasive species (Goldsmit et al. 2014; Lacoursière‐Roussel et al. 2018). Increasing the use of eDNA technologies, which enable whole ecosystem assessments, will be pivotal for increasing the scale and scope of monitoring surveys in Arctic Canada and has already improved detection sensitivity for potential invasive species and species northward migrations in other Arctic regions (Jensen et al. 2023; van den Heuvel‐Greve et al. 2021). These surveys will be vital for providing current snapshots of community composition against which future ecosystem changes resulting from invasive species establishing and/or climate change can be evaluated.

To date, there have been very few large‐scale biodiversity surveys across Arctic Canada, limiting our understanding of ecological connectivity and how the ecosystem will respond to stressors, such as climate change or increased human activities (Archambault et al. 2010; Darnis et al. 2022; Jacquemot et al. 2024). eDNA metabarcoding studies have so far largely been constrained to small spatial scales, for example, in ports (Brown et al. 2016; Lacoursière‐Roussel et al. 2018; Leduc et al. 2019). However, given the current rates of warming and globalization, increased biodiversity surveillance across the whole of Arctic Canada is needed to dramatically improve our understanding of spatiotemporal differences in community composition. In this study, we utilize ships of opportunity to undertake eDNA sampling along the majority of the southern route of the NWP, one of the shipping routes experiencing the highest increases in shipping activity within Arctic Canada, and thus a potential risk hotspot for invasive species introductions. Our aims include providing a snapshot of biodiversity in this area, assessing spatial differences in community composition, and identifying any potential invasive species.

## 2 Methods

### 2.1 Sample Collection

**FIGURE 1:**
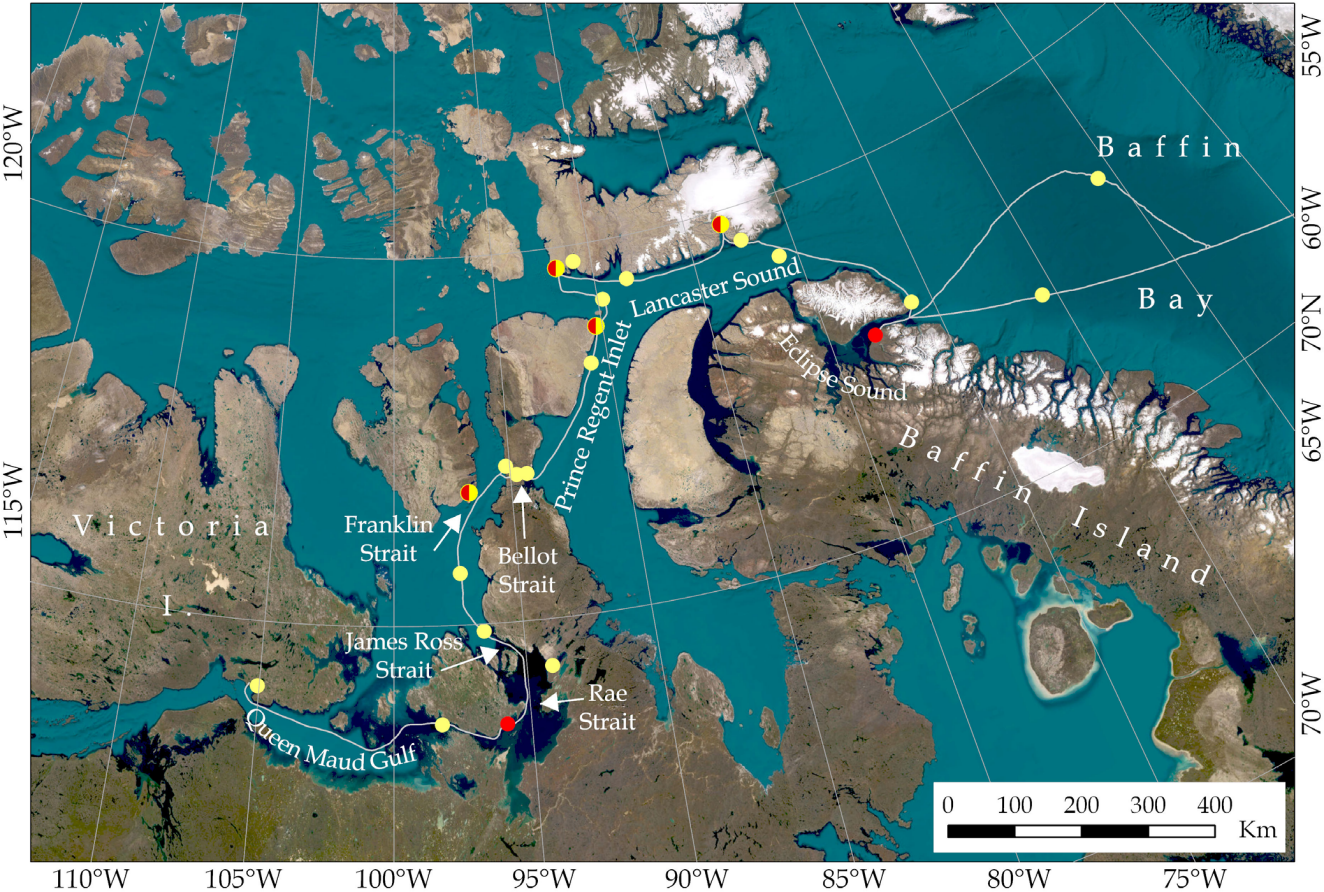
Map showing the locations of environmental DNA sampling points along the Northwest Passage Sea Route in Arctic Canada. Red circles represent fixed samples taken at points of interest where cruise ships regularly visit, yellow circles represent underway samples whilst the ship in transit and half red half yellow circles indicate sample locations where both fixed and underway samples were collected.

Sampling took place on board the MS Fram (HX Hurtiguten Expeditions) in August and September 2023 along the Northwest Passage between Cambridge Bay and Baffin Bay (Figure 1). Samples were collected at fixed points along the route when the cruise ship stopped at points of interest (natural or historical sites), as well as underway samples whilst the ship was in transit. For the fixed point samples, 1 L seawater samples were collected in triplicate using a 1.7 L Niskin bottle lowered to 5 m depth to be consistent with the underway sample collection depth via the seawater inlet valve in the hull. Seawater samples were transferred from the Niskin bottle to sterile aluminium foil ‘Bag in Box’ containers for transport and storage prior to filtering. Underway seawater samples were collected in triplicate directly into ‘Bag in Box’ containers from a hose connected to the sea chest. At four locations, we collected both fixed and underway samples to confirm that the sea chest was delivering a fresh supply of seawater and to detect any organisms potentially living in the ship's pipework. To reduce contamination, the Niskin bottle was soaked in 5% bleach in a 1:1 dilution for half an hour and then rinsed thoroughly with tap water prior to each deployment. We also collected tap water blanks in the Niskin bottle before sampling each set of triplicate fixed samples, and directly into ‘Bag in Box’ containers for underway samples. In total, we collected 27 seawater samples (81 samples including replicates), from six fixed point locations and 21 underway locations, along with 25 field blanks (as described above; Table S1).

### 2.2 Sample Filtration

Filtration occurred as soon as possible after sample collection, with all samples filtered within 24 h. Samples were filtered through cellulose nitrate filters with 0.8 μm pore size using Nalgene reusable analytical funnels (Thermo Scientific) and the Fisherbrand FB70155 vacuum pump. Immediately after filtration, filter papers were wrapped in aluminum foil and stored at −20°C.

### 2.3 Molecular Analyses

DNA extractions and PCR amplifications took place in a dedicated pre‐PCR molecular laboratory, where all surfaces were thoroughly cleaned with a 4.7% bleach solution and double distilled water before use. Laboratory equipment, i.e., pipettes and sample blocks, were exposed to ultraviolet light for 30 min between sample preparations. DNA was extracted with the Qiagen DNeasy PowerSoil Pro Kit following the manufacturer's protocol. DNA was amplified with two different primer sets, targeting 18S rRNA and COI, that characterize Arctic eukaryotic communities well (Jacquemot et al. 2024; Lacoursière‐Roussel et al. 2018). We used 1391F (5′‐GTACACACCGCCCGTC‐3′) and EukBr (5′‐TGATCCTTCTGCAGGTTCACCTAC‐3′) to amplify a 260 bp fragment from the 18S rRNA V9 region (Amaral‐Zettler et al. 2009; Stoeck et al. 2010), and mlCOIintF‐XT (5′‐GGWACWRGWTGRACWITITAYCCYCC‐3′) and jgHCO2198 (5′‐TAIACYTCIGGRTGICCRAARAAYCA‐3′) to amplify a 313 bp fragment from the mitochondrial COI region (Leray et al. 2013; Wangensteen et al. 2018). We followed a two‐step PCR approach for both primer sets, whereby sequences homologous to the Illumina sequencing adapters were appended to the 5′ end of the forward (5′‐TCGTCGGCAGCGTCAGATGTGTATAAGAGACAG‐3′) and reverse (5′‐GTCTCGTGGGCTCGGAGATGTGTATAAGAGACAG‐3′) primers for the first PCR, and samples were uniquely indexed with Nextera XT adapters in the second PCR. For 18S, PCR reactions were 20 μL consisting of 1 μL template DNA, 1.25 units GoTaq Hot Start Polymerase (Promega), 5× Green GoTaq Flexi Buffer (Promega), 2 mM MgCl_2_ (Promega), 0.2 mM dNTPs (Promega), 1.6 μM mammal blocking primer (5′‐GCCCGTCGCTACTACCGATTGG/ideoxyI//ideoxyI//ideoxyI//ideoxyI//ideoxyI/TTAGTGAGGCCCT/3SpC3/‐3′), 0.2 μM of each the forward and reverse primer and UltraPure distilled water (Invitrogen). Thermocycling conditions consisted of an initial denaturation at 94°C for 3 min, followed by 35 cycles at 94°C for 45 s, 65°C for 15 s, 57°C for 30 s, and 72°C for 90 s, and a final extension at 72°C for 10 min. For COI, PCR reactions were 25 μL consisting of 2 μL template DNA, 1.25 units GoTaq Hot Start Polymerase (Promega), 5× Green GoTaq Flexi Buffer (Promega), 1 mM MgCl_2_ (Promega), 0.2 mM dNTPs (Promega), 0.5 μg/μL bovine serum albumin (Thermo Scientific), 0.4 μM of each the forward and reverse primer and UltraPure distilled water (Invitrogen). Thermocycling conditions consisted of an initial denaturation at 95°C for 10 min, 35 cycles of 94°C for 1 min, 45°C for 1 min, and 72°C for 1 min, and a final elongation at 72°C for 5 min. Amplicons were purified and primer dimers were removed using AppMag PCR Clean Up Beads (0.9× ration; Appleton Woods). The final sequencing libraries for both 18S and COI were generated using a second PCR in which Nextera XT indexed adaptor sequences were used as primers, so that each sample and sample triplicates were uniquely indexed. The PCR reaction consisted of 20 ng of the pooled amplicons diluted with nuclease‐free water to 5 μL input volume, 12.5 μL NEBNext Q5 Hot Start HiFi PCR Master Mix, 2.5 μL each of the appropriate Nextera XT Index Primer 1 and Primer 2. Thermocycling conditions included an initial denaturation at 95°C for 3 min, followed by eight cycles of 95°C for 30 s, 55°C for 30 s, 72°C for 30 s, and a final hold at 72°C for 5 min. The products were then purified again, as above, to remove adapter dimers and free adapter oligos and checked for the presence of correctly formed libraries by running on a D1000 tape of a Tapestation 4200 followed by fluorometric quantification using the Quant‐iT PicoGreen dsDNA Assay. Libraries were then combined into equimolar pools individually for 18S and COI amplicons and sequenced separately on a MiSeq 250 bp paired‐end lane with v2.0 chemistry, 7 pM loading concentration, and 30% PhiX control library to overcome low diversity pools associated with amplicon sequencing.

### 2.4 Bioinformatic Analyses

Primers and adapters were trimmed from sequences with CUTADAPTv4.6 (Martin 2011), and only reads longer than 100 or 150 bp were retained for 18S and COI, respectively. Sequence quality control checks were carried out with FastQCv0.12.1 (https://www.bioinformatics.babraham.ac.uk/projects/fastqc/). The DADA2 workflow was implemented to remove poor quality reads and chimeras, denoise the data, dereplicate identical reads, and generate Amplicon Sequence Variants (ASVs) (Callahan et al. 2016). Taxonomic assignment was carried out using BlastN against the SILVA database for 18S and the Bold database for COI. The BLAST search recorded the 10 best matches ranked by the *e*‐value, with a cutoff threshold of 1e‐25. Sequences that had < 98% similarity to a sequence in the reference databases were manually blast searched against the NCBI nucleotide database, and either assigned to a taxon if within the 98% threshold or removed from the dataset. We chose a stringent similarity threshold (98%) as the primary aim of this study was to identify invasive species, so we required accurate species‐level assignments (Bonin et al. 2023). In instances where sequences had multiple ‘best matches’, these were checked manually and assigned to the correct native species if available. If multiple closely related native species matched to an ASV within the similarity threshold, then taxonomic assignments were collapsed to the genus or lowest common taxonomic resolution possible. Full taxonomic ranks for the final taxon assignment were obtained from the World Register of Marine Species (WORMS) (Ahyong et al. 2024). We predominantly used the Arctic Register of Marine Species (ARMs; https://www.marinespecies.org/arms/index.php) and Arctic Protist Flora (https://arctic‐protist‐flora.scrol.net/) databases, supplemented with primary literature, to assess whether a species was native to the Arctic (Sirenko et al. 2024). These databases include flora and fauna from across the Arctic, as no regional publicly accessible databases for the study area exist at present.

### 2.5 Community Analyses

ASVs detected in the field, DNA extraction and PCR blanks were used to filter out contamination across the sample handling workflow. Negligible contamination for the COI primer set was detected across the extraction (0–18 reads per blank, 0.001% total COI sample reads), PCR (0–43 reads per blank, 0.003% total COI sample reads) and field blanks (0–464 reads per sample, 0.05% total COI sample reads), corresponding to seven highly abundant ASVs across COI samples from field collected material, so no further contamination control was applied as the contamination levels were so low (Lacoursière‐Roussel et al. 2018). Contamination was more prevalent in the 18S dataset, corresponding to 85 ASVs and 5% of the overall reads obtained. Therefore, we consecutively subtracted the sample‐specific read abundance from field blanks for each ASV, followed by the average read abundance in extraction and PCR blanks (Boyse et al. 2024). Twenty‐three 18S ASVs were unique to control samples and a further two ASVs, belonging to *Fragilariopsis cylindrus* and *Picomonas*, had no reads left in field samples following contamination control.

Initial eukaryotic community composition characterizations and data visualizations were carried out with the Phyloseq (version 1.46.0) and Metacoder (version 0.3.7) R packages (Foster et al. 2017; McMurdie and Holmes 2013). To analyze spatial variation in community composition, we partitioned the data into different marine regions according to distinct water bodies or geographical separation by islands. We assessed spatial differences in alpha and beta diversity across the marine regions with the Vegan (version 2.6.6.1) R package (Dixon 2003). For these community analyses, we converted the data into an ASV‐specific index to compare abundances for each ASV spatially across samples to account for amplification biases differing among taxa, equivalent to Wisconsin double‐standardization (Djurhuus et al. 2020; Kelly et al. 2019). Initially, read counts were converted into proportions, and the resulting proportions were scaled into an index between 0 and 1 for each ASV by dividing the largest proportion for each ASV from all other occurrences of the ASV across samples (Kelly et al. 2019). We calculated alpha diversity per sample with the Shannon‐Weiner index. We compared whether alpha diversity significantly differed across marine regions with analysis of variance (ANOVA) and Tukey's HSD test to assess pairwise differences if the data were normally distributed, or the Kruskal–Wallis test and pairwise Wilcox test alternatively. We evaluated whether beta diversity differed between marine regions with non‐metric multidimensional scaling (NMDS) of Bray–Curtis dissimilarity distances. Significant differences in beta diversity were assessed using permutational multivariate analysis of variation (PERMANOVA) and further multilevel pairwise comparisons were made with the pairwiseAdonis R package (version 0.4.1) (Martinez 2017).

We obtained environmental variables that are important for determining species distributions to assess environmental conditions against previously known environmental tolerance limits for any invasive species detected. The General Bathymetric Chart of the Oceans (GEBCO) 2024 bathymetry grid was downloaded at a 0.004° × 0.004° (GEBCO Compilation Group 2024). Daily rasters for sea surface temperature (SST; https://doi.org/10.48670/moi‐00130) and sea water salinity (https://doi.org/10.48670/moi‐00001) were obtained at a 0.05° × 0.05° and 0.08° × 0.0625° resolution, respectively, from the E.U. Copernicus Marine Service Information (Melsom et al. 2012; Sakov et al. 2012). SST and salinity could not be determined for 14 and 18 of the sample sites, respectively, as they were out of range of the environmental raster grids.

We used AIS data obtained from exactAIS, operated by Spire Global (now Kpler), to assess ship traffic passing within 10 km of the sample sites up to 2–12 h prior to sample collection to understand the likelihood of detecting hull fouling on other vessels. This dataset captures dynamic ship movements, including MMSI, timestamp, position, speed, and course, and was delivered in standardised formats suitable for spatial and temporal analysis.

## 3 Results

### 3.1 General Overview of Eukaryotic Community Composition

Following bioinformatic analyses and taxonomic filtering, we recovered 1,733,376 COI sequences corresponding to 126 ASVs at the species level or the lowest taxonomic level of assignment possible, and 4,236,720 sequences belonging to 391 unique ASVs for 18S rRNA (Table 1; Data S1). For all samples where an ASV was detected, it was most common for an ASV to only be detected in one field replicate (65%), while an ASV being detected in two (19%) or three (15%) replicates was less common. ASVs that were detected in all three field replicates were also in the top 10% of the most abundant ASVs detected in regard to total reads across all samples.

**TABLE 1:** The total number of reads across seawater samples assigned to different kingdoms and the number of taxonomically unique ASVs detected from the COI and 18S primer sets.

Kingdom	COI—total reads (% of reads)	COI—ASVs detected	18S—total reads (% of reads)	18S—SVs detected
All	1,717,640	126	3,625,040	365
Animalia	836,888 (48.7%)	69	895,924 (25%)	75
Chromista	624,698 (36.4%)	49	2,645,195 (73%)	224
Plantae	253,631 (14.8%)	7	37,284 (< 1%)	40
Protozoa	2432 (0.1%)	1	46,637 (< 2%)	26

**FIGURE 2:**
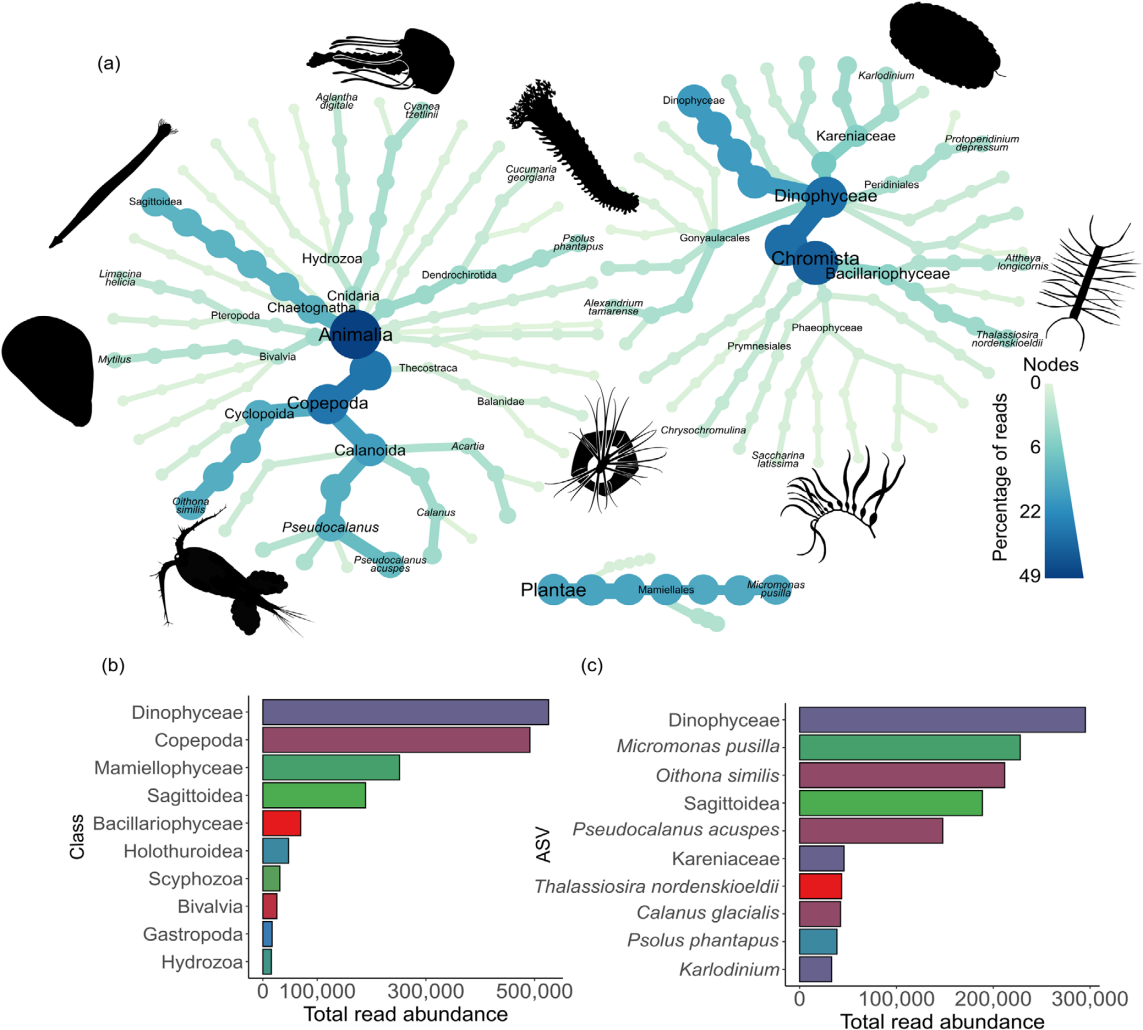
(a) Heat trees showing diversity recovered with the COI primer set for ASVs with > 500 reads. Bar charts showing (b) the top 10 most abundant Classes and (c) ASVs at species level or the highest taxonomic resolution possible based on the total number of reads across all samples with the COI primer set. The color of the ASVs in (c) correspond to the class that the ASV belongs to in (b).

Almost half (48.7%) of all COI reads were assigned to the Animalia kingdom, with the majority of Animalia reads belonging to the Copepoda (58.7%) and Sagittoidea (arrow worms; 22.6%) classes (Figure 2). Thirteen different species of copepods were detected, with *Oithona similis* , *Pseudocalanus acuspes* , and *Calanus glacialis* having the most abundant read counts across all samples. Thirty different ASVs belonging to the Sagittoidea class were detected, but none could be resolved to a lower taxonomic level. Chromista was the second most abundant kingdom, accounting for 36.4% of total COI reads, with Dinophyceae (84%) and Bacillariophyceae (11%) classes (11%) dominating Chromista read counts. Fifty‐nine percent of unique ASVs assigned to the Dinophyceae class could not be resolved to a lower taxonomic level, although the remaining Dinophyceae ASVs were resolved to lower taxonomic levels, including 11 species, with the Kareniaceae family and *Karlodium* genus being the most abundantly detected. Five species from the Bacillariophyceae class were detected, with *Thalassiosira nordenskioeldii* (62%) and *Attheya longicornis* (36%) accounting for the majority of reads. 14.8% of COI reads were assigned to members of the Plantae kingdom, of which *Micromonas pusilla* from the Mamiellophyceae class contributed to 89.8% of reads.

**FIGURE 3:**
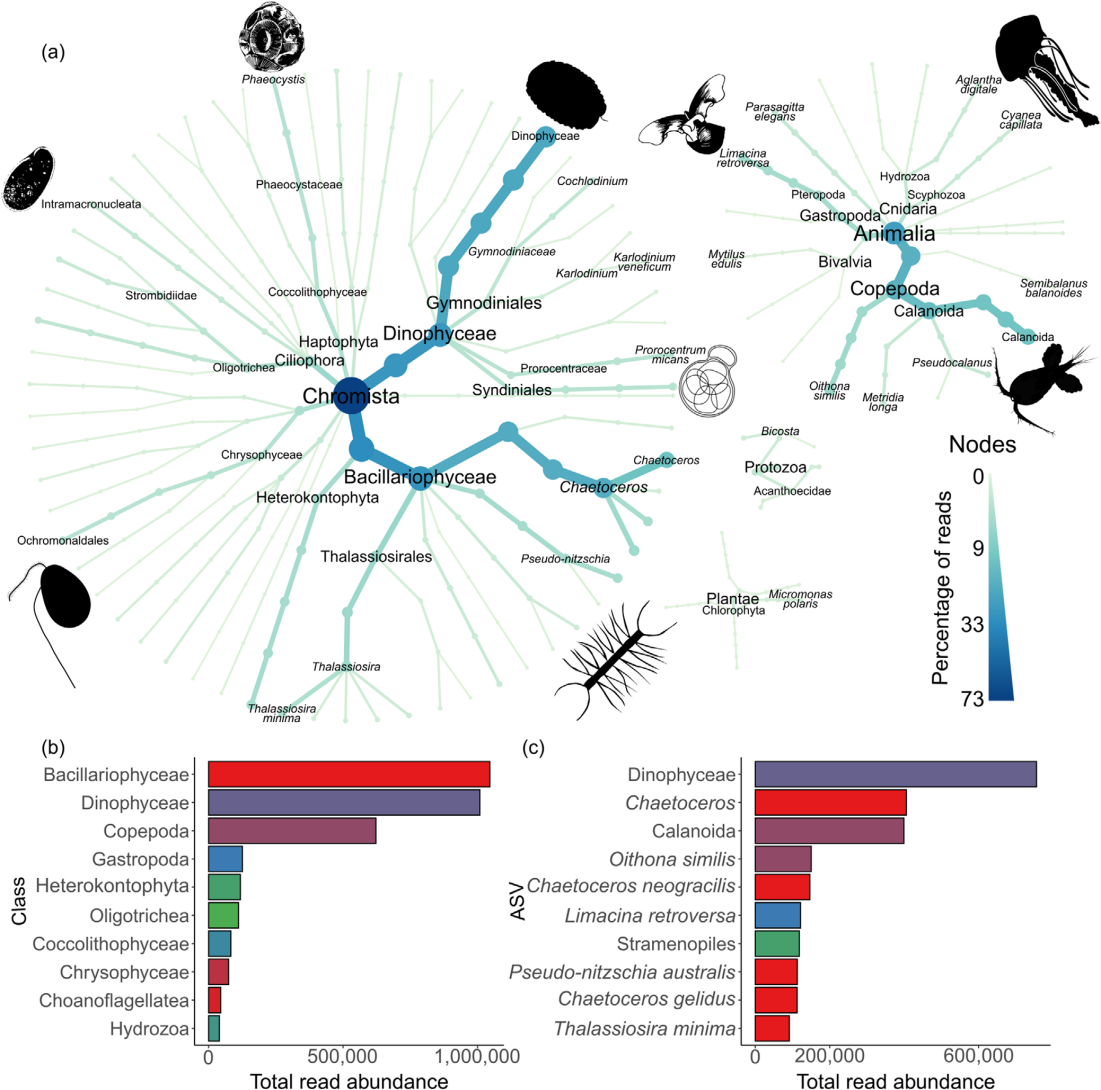
(a) Heat trees showing diversity recovered with the 18S rRNA primer set for ASVs with > 5000 reads. Bar charts showing (b) the top 10 most abundant Classes and (c) ASVs at the species level or the highest taxonomic resolution possible based on the total number of reads across all samples with the 18S primer set. The color of the ASVs in (c) correspond to the class that the ASV belongs to in (b).

18S reads were dominated by ASVs belonging to the Chromista Kingdom, contributing to 73% of the total reads obtained (Figure 3). The Dinophyceae class and *Chaetoceros* genus from the Bacillariophyceae class made up over 50% of reads assigned to the Chromista Kingdom. Other abundant ASVs from the Chromista Kingdom included Stramenopiles and the diatoms *Pseudo‐nitzschia australis* and *Thalassiosira minima* (and other members of this genus). Animalia reads contributed to 24% of total 18S reads, with the Copepoda class contributing to over 50% of Animalia reads. Similar to the COI dataset, the copepod *O. similis* was the most abundant Animalia ASV designated at the species level, while the majority of other copepod ASVs were assigned to the Calanoida order but could not be identified to the species level. Gastropoda was the second most abundant Animalia class, with the pteropod *Limacina retroversa* contributing to 97% of Gastropoda reads.

### 3.2 Spatial Variation in Community Composition

**FIGURE 4:**
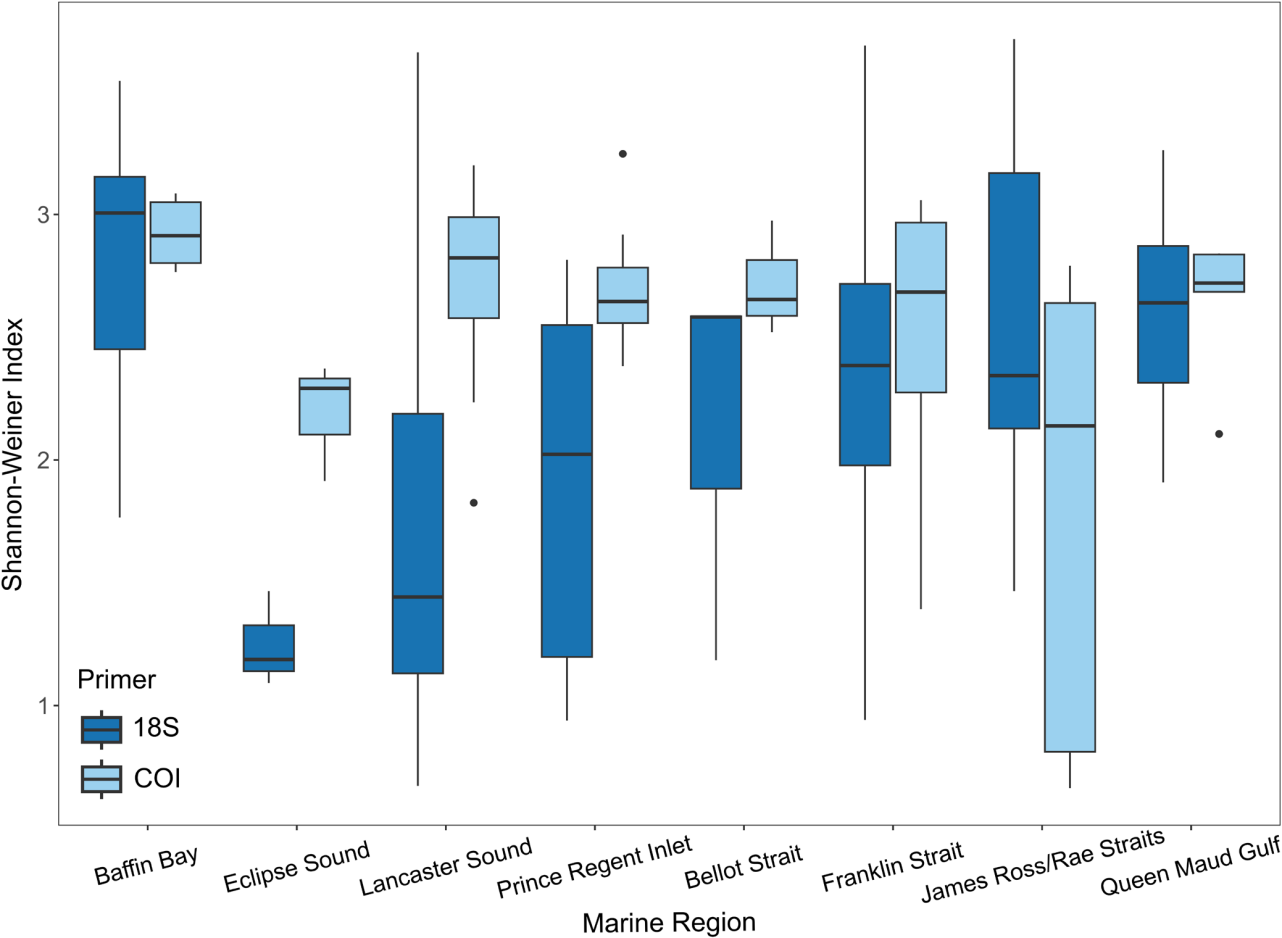
Alpha diversity estimated with the Shannon‐Weiner Species Diversity Index for 18S and COI‐derived communities across different marine regions surveyed.

**FIGURE 5:**
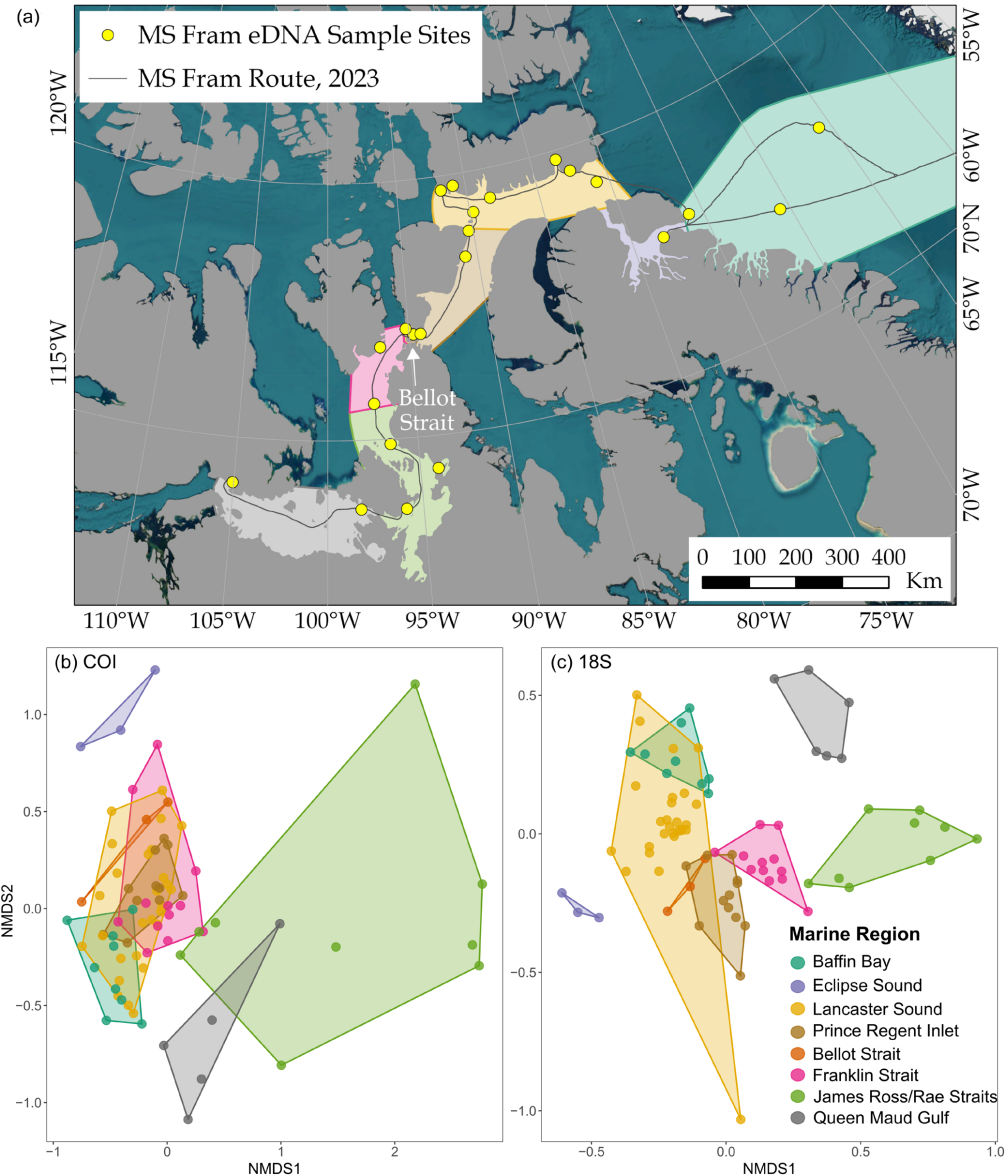
(a) Map showing the environmental DNA sampling points within distinct marine regions along the survey route. Non‐metric multidimensional scaling (NMDS) plots with Bray–Curtis dissimilarity showing dissimilarity of community composition across the different marine regions derived from the (b) COI (*k* = 2, stress = 0.2) and (c) 18S rRNA (*k* = 2, stress = 0.19) primer sets for Arctic eukaryotes.

Alpha diversity significantly differed between marine regions for both the COI (Kruskal–Wallis *χ* ^2^ = 21.78, df = 7, *p* < 0.01) and 18S primer sets (ANOVA, df = 7, *F* = 3.431, *p* < 0.01). Baffin Bay had the highest alpha diversity across all marine regions, and this was significantly higher than alpha diversity in Lancaster Sound for 18S (Tukey HSD, *p* < 0.05), and Eclipse Sound, James Ross/Rae Straits, and Prince Regent Inlet with COI (pairwise Wilcoxon rank‐sum test < 0.05). Eclipse Sound had the lowest alpha diversity with 18S, whilst James Ross/Rae Straits and Eclipse Sound had the lowest alpha diversity with COI (Figure 4). Beta diversity also significantly differed between marine regions with both the COI (*adonis2*: df = 7, *F* = 3.77, *R* ^2^ = 0.27, *p* < 0.001) and 18S (*adonis2*: df = 7, *F* = 4.49, *R* ^2^ = 0.3, *p* < 0.001) primer sets. NMDS shows a distinction between James Ross/Rae Straits and Queen Maud Gulf, the two regions at the lowest latitude, compared with other marine regions (Figure 5). Beta diversity for James Ross/Rae Straits differed significantly from Baffin Bay, Lancaster Sound, Prince Regent Inlet, and Franklin Strait, and likewise for Queen Maud Gulf from Lancaster Sound, Prince Regent Inlet, and Franklin Strait with the COI primer set (pairwise adonis, *p* < 0.05). With the 18S marker, Baffin Bay beta diversity is significantly different from Lancaster Sound, Prince Regent Inlet, and Franklin Strait, and Lancaster Sound significantly differs from Queen Maud Gulf. Samples taken at Pond Inlet within Eclipse Sound appear to have distinct beta diversity compared with all other regions (Figure 5) but only significantly differ from Lancaster Sound with the COI primer set, likely due to small sample size (three replicates).

**FIGURE 6:**
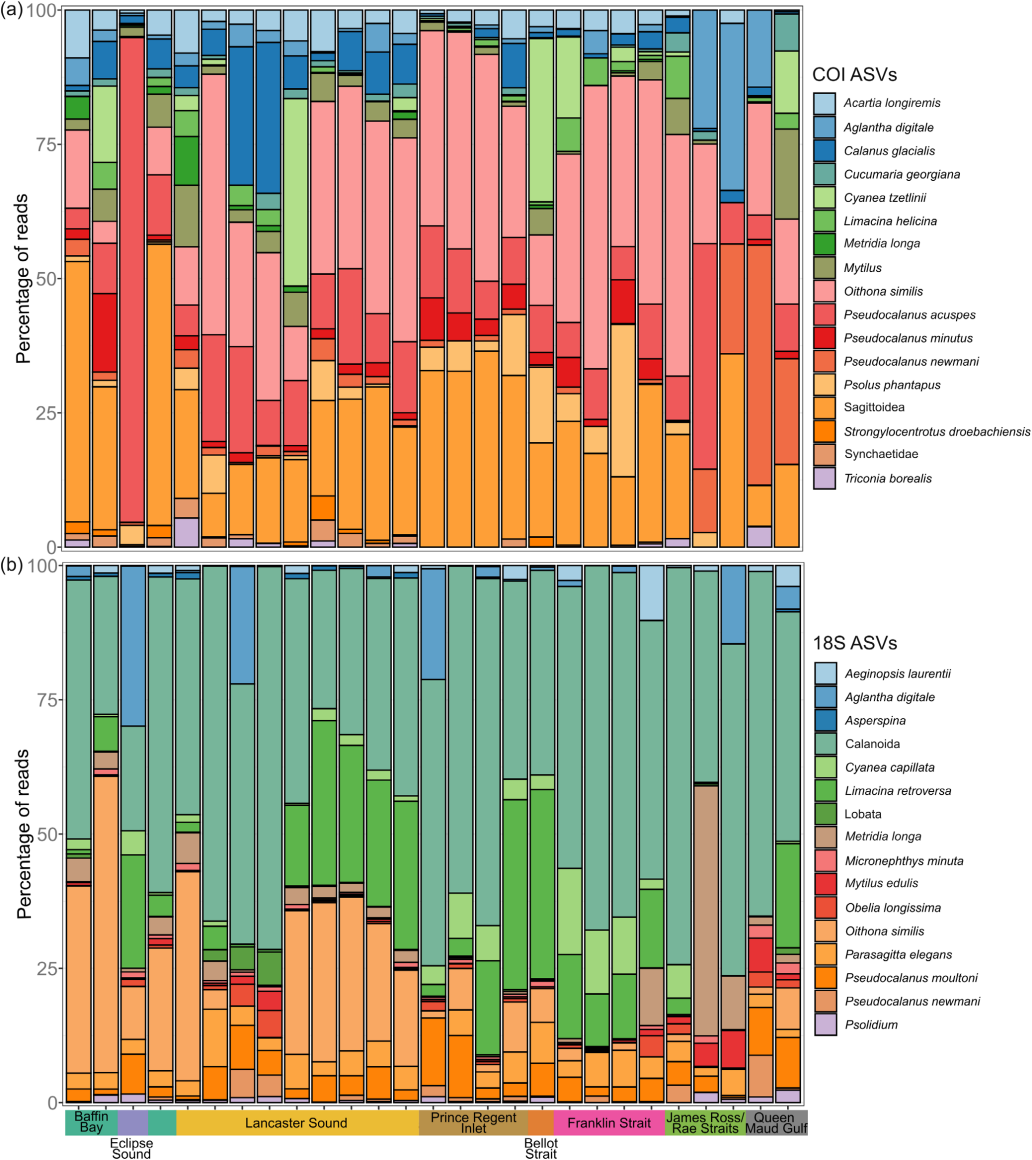
Stacked bar charts showing Animalia detected with the (a) COI primer set and (b) the 18S rRNA primer set with > 3000 reads. Samples are ordered in chronological order along the survey vessel's route and separated into marine regions to visualize spatial differences in ASV abundance.

**FIGURE 7:**
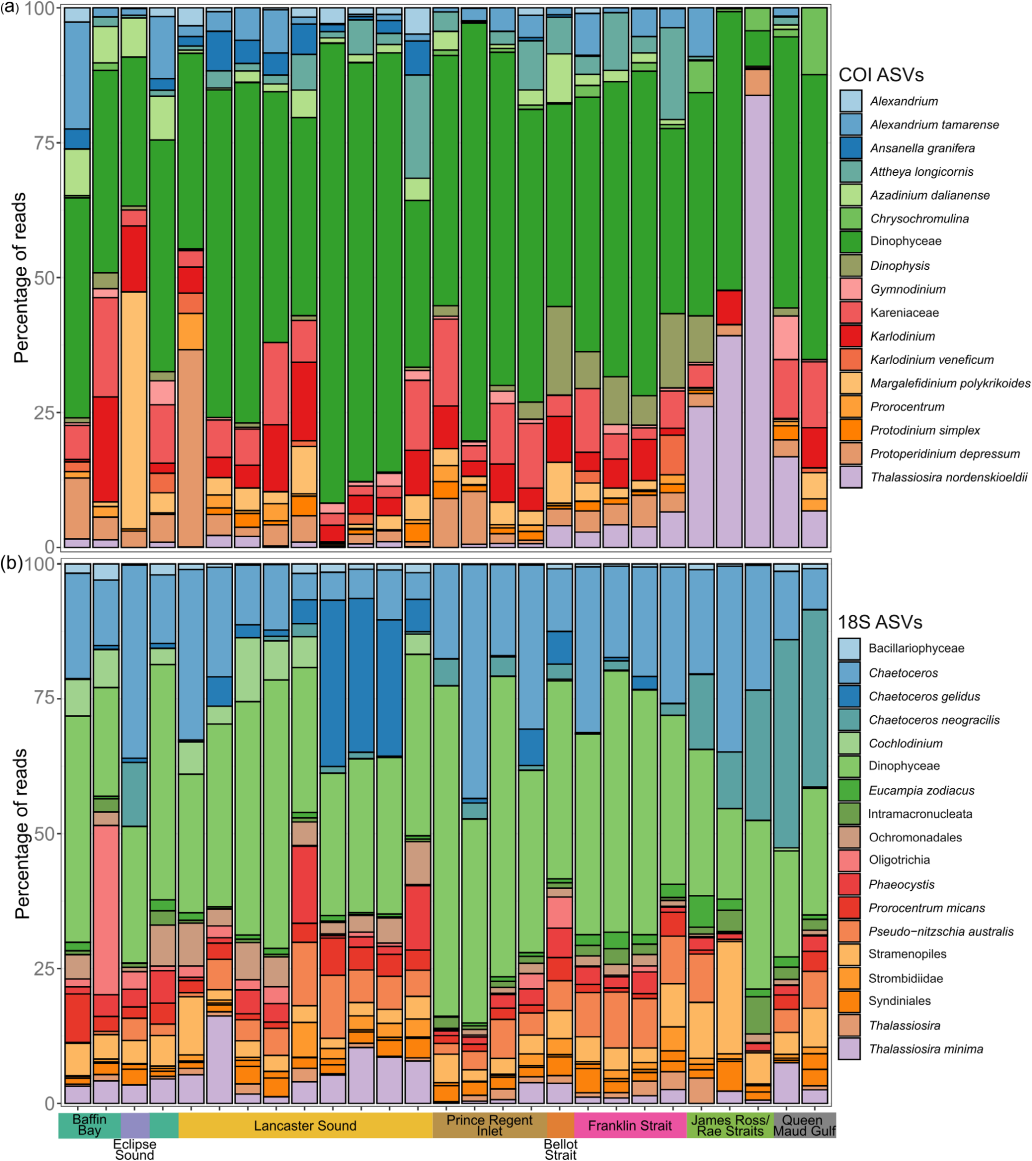
Stacked bar charts showing Chromista detected with the (a) COI primer set with > 5000 reads and (b) the 18S rRNA primer set with > 20,000 reads. Samples are ordered in chronological order along the survey vessel's route and separated by marine region to visualize spatial differences in ASV abundance.

In general, the dominant taxonomic groups remained consistent across the survey area and between the 18S and COI primer sets (Figures 6 and 7). For Animalia across both primer sets, copepods contributed to the largest proportion of read counts, although the Calanoida order was more abundant with 18S and *O. similis* with COI. This difference stems from the Calanoida order being resolved to species level with COI and so represented by 11 species from four genera (*Acartia*, *Calanus*, *Metridia*, *Pseudocalanus*). With 18S, *O. similis* was more prevalent in Baffin Bay and Lancaster Sound, whereas *O. similis* was detected in consistently high abundance along the survey route with COI (Figure 6). Other copepods showed peaks in abundance in certain regions; for example, *Metridia longa* was most abundant in James Ross Strait with 18S, *Calanus glacialis* in Baffin Bay and Lancaster Sound with COI, and *Pseudocalanus newmani* in the Rae Strait and Queen Maud Gulf with COI. The sea snail, *Limacina*, was more prevalent in Prince Regent Inlet and Bellot Strait with the 18S primer set, whereas *Limacina* contributed to a smaller proportion of COI read counts, and abundance varied across the survey area (Figure 6). For Chromista across both primer sets, the Dinophyceae class consistently contributed the greatest proportion of reads across the survey route (Figure 7). The *Chaetoceros* diatom genus also contributed to a large proportion of read counts for 18S consistently across the survey route, with a peak in abundance for *Chaetoceros gelidus* in western Lancaster Sound and for *Chaetoceros neogracilis* in Rae Strait and Queen Maud Gulf. The most abundant diatoms detected with COI also showed peaks in abundance, with *A. longicornis* most prevalent in western Lancaster Sound, Prince Regent Inlet, and Franklin Inlet and *Thalassiorsira nordenskioeldii* in the James Ross/Rae Straits and Queen Maud Gulf (Figure 7).

### 3.3 Potential Invasive Species Detections

**FIGURE 8:**
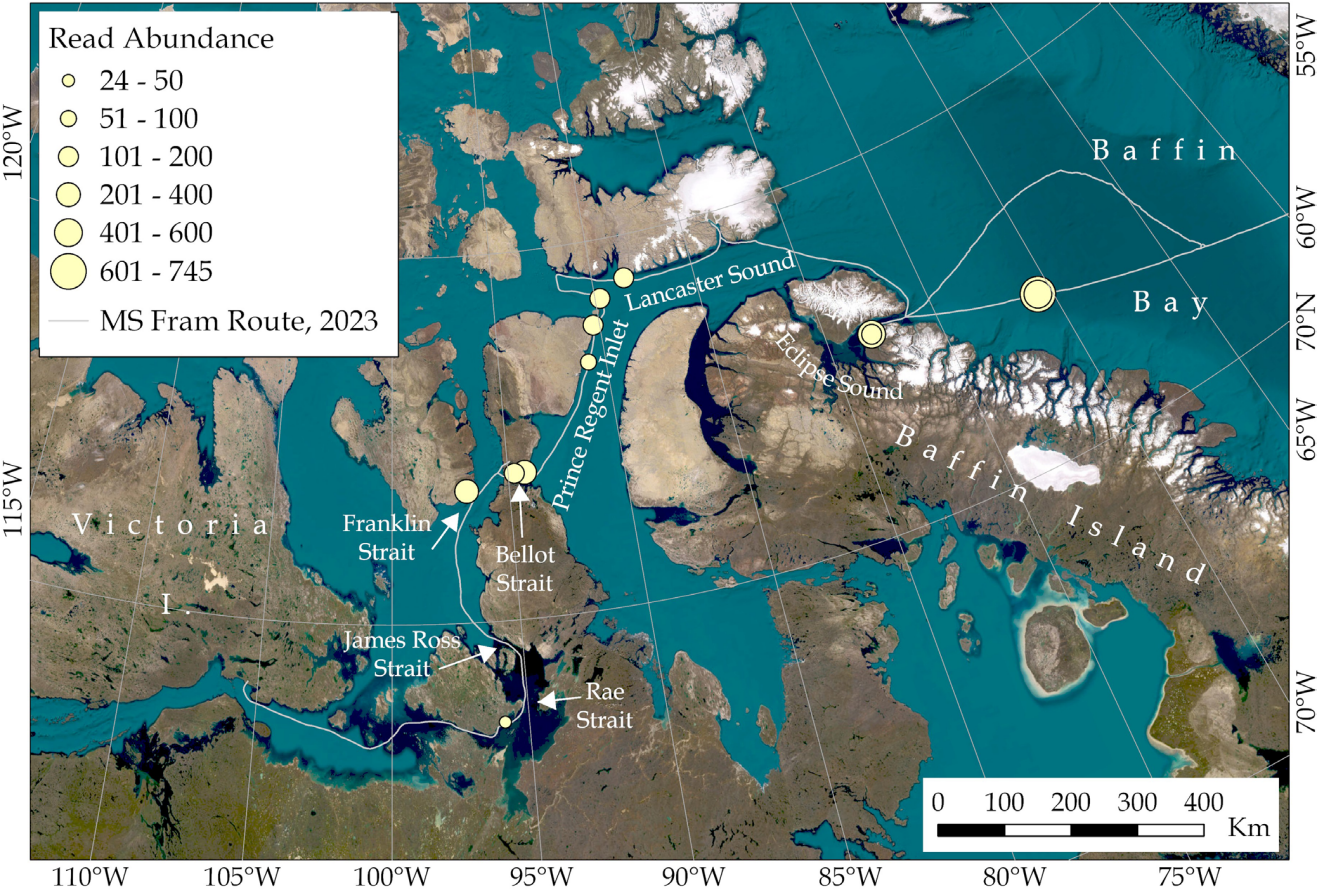
Map showing locations (yellow circles) where the invasive bay barnacle (*Amphibalanus improvisus*) was detected with environmental DNA metabarcoding. The size of the circle corresponds to the number of read counts assigned to *A. improvisus* .

Identification of potentially invasive species was carried out using the Arctic Register of Marine Species (ARMs; https://www.marinespecies.org/arms/) and the Arctic Protist Flora (https://arctic‐protist‐flora.scrol.net/) as baseline databases for validated Arctic species identifications, alongside published analyses of high‐risk potential invasive species in the Arctic (Goldsmit et al. 2014, 2018). Using these datasets, one potential high‐risk invasive species, the Bay Barnacle (*Amphibalanus improvisus*), was detected in this study with the COI primer set only. *A. improvisus* was detected at 10 different sampling locations, including four fixed sample replicates and eight underway sample replicates, spanning much of the survey area between Gjoa Haven and Baffin Bay. In terms of representation in replicates at field sites, *A. improvisus* was detected in one field replicate at eight sampling locations and two field replicates in two sampling locations. A total of 2914 reads across three ASVs were assigned to *A. improvisus* with 99%–100% percent identity similarity, ranging between 24 and 745 reads in any individual sampling replicate. The highest read counts were obtained from the east of the survey area near Pond Inlet and in Baffin Bay (Figure 8). According to the Arctic Register of Marine Species (ARMS), there are five native barnacle species in the Arctic: *Balanus balanus* , *Balanus crenatus* , *Balanus rostratus* , *Chirona hameri* , and *Semibalanus balanoides* , with no native members of the *Amphibalanus* genus. Only one of the native species, *S. balanoides* , was detected in our survey, with both COI and 18S primer sets. The native barnacle was detected by at least one of the primer sets at 9 out of 10 locations where the invasive species was detected, with the highest read abundance also being in Baffin Bay. COI reference sequences are available for all five native barnacles in both NCBI and Bold databases, so we aligned these sequences with the three *A. improvisus* ASVs to compare sequence similarity. *B. balanus* and *S. balanoides* sequences were most similar to *A. improvisus* with 84% percent identity, with 83% for *B. crenatus* , 81% for *B. rostratus* , and 80% for *C. hameri*. Sample locations where *A. improvisus* was detected had variable environmental conditions, including depth between 5 and 1570 m (average 254 ± 479 m SD), salinity between 29.1 and 31.35 ppt (average 30.27 ± 0.99 SD) and sea surface temperature between 1.96°C and 3.17°C (average 2.57°C ± 0.4 SD). At the exact time of sampling, additional ships in the area were detected within 10 km of three sampling locations: Pond Inlet (two ships), Croker Bay (one ship) and Cambridge Bay (one ship). Up to 2 h prior to sampling the water column, two additional ships were detected within 10 km of Pond Inlet, and three additional ships at Croker Bay. Up to 12 h prior to sampling taking place, the number of ships within Pond Inlet and Croker Bay remained the same, with one additional ship detected at Cambridge Bay as well. At all other sample locations, no ships were detected within a 10 km radius of the sampling sites within 12 h prior to sample collection.

Six further non‐Arctic species were detected with the COI primer set. Five of these species (*Anatoma euglypta*, *Benthalbella elongata* , *Gymnoscopelus nicholsi* , *Heterocucumis steineni* , *Paradiplospinus*) have known distributions in Antarctica, so they were likely detected because of laboratory cross‐contamination. Although every effort was made to reduce potential contamination risk, the molecular laboratory where the extractions and PCRs were performed conducts research on Antarctic fisheries, gastropod phylogeny, and uses *H. steineni* as a model species; hence their likely accidental representation in this sample set. The remaining species, the tiger pistol shrimp ( *Alpheus bellulus* ), has a tropical distribution, so it is highly unlikely to truly be present in the Arctic (Data S1).

Twelve species were detected with 18S that had not previously been recorded in the Arctic. The gumboot chiton, *Cryptochiton stelleri* , was detected at one underway station with 300 reads. This species is native to rocky intertidal habitats in the eastern Pacific, including Japan, California, and Alaska, and has not previously been reported as an invasive species (Lord 2011; McIntire and Bourdeau 2020). We recommend that further research would be necessary to confirm this species presence and potential impact in the Arctic prior to classifying the gumboot chiton as a potential invasive species. *Acineta* represents contamination from a common terrestrial plant genus, orchids, whilst the athecate hydroid, *Fabienna spaerica*, has only been recorded in the southwest Pacific, so it is highly unlikely to be present in the Arctic. The nine remaining species ( *Carinoma tremaphoros* , *Chaenea vorax*, *Eupogodon spinellus*, *Gymnophyrs*, *Halocynthia roretzi*, *Parabirojimia similis*, *Phaeostrophion irregulare* , *Phallusia mammillata* , *Pseudomystides limbata* ) were only detected at one or two locations, and with less than 50 reads, largely representing microscopic organisms. Their lack of previous detection is likely due to the paucity of Arctic biodiversity sampling, which is highlighted by the fact that since the original draft of this manuscript (which detailed 25 non‐Arctic species detected with 18S), 13 have now been identified in a very recently released metagenomic dataset from water sampling around Nova Scotia and New Brunswick (Krumhansl et al. 2025).

## 4 Discussion

We provide a current snapshot of eukaryotic community composition during the ice‐free season along the Northwest Passage, one of the shipping routes that has seen the largest increases in shipping traffic in Arctic Canada. Our study demonstrates the effectiveness of eDNA metabarcoding as an Arctic monitoring tool for detecting potential invasive species and characterizing current eukaryotic community composition, particularly zooplankton and phytoplankton, as would be expected for water column sampling (Cleary et al. 2025). We report the first known detections of a highly invasive species, the bay barnacle ( *A. improvisus* ), in Arctic Canada, most likely as a direct consequence of increased shipping traffic coupled with warming ocean temperatures.

### 4.1 Community Composition

Dominant species in both the Animalia and Chromista kingdoms from our eDNA‐based survey correlate well with previous net sampler studies within the region and across broader Arctic communities. *O. similis* was the most abundant copepod ASV assigned at species level with both the 18S and COI primer sets (Figures 2 and 3), aligning with previous surveys that have also found *O. similis* to be the most abundant copepod within the Canadian Arctic Archipelago, as well as more broadly across the Arctic including the Barents Sea, Beaufort Sea, Hudson Bay, Labrador Sea, Canadian Basin, Greenland Sea, and central Arctic Ocean (Ashjian et al. 2003; Auel and Hagen 2002; Darnis et al. 2022; Dvoretsky and Dvoretsky 2013; Hopcroft et al. 2005; Møller et al. 2006). Whilst the small‐bodied *O. similis* (< 0.45 mm prosome length) usually dominates absolute abundance estimates, larger copepods such as *Calanus* spp. and *M. longa* (< 7 mm prosome length) contribute towards a larger proportion (< 80%) of overall zooplankton biomass (Darnis et al. 2022; Rutzen and Hopcroft 2018). Higher read counts obtained for *O. similis* are therefore surprising given previous studies linking eDNA signals with biomass (Di Muri et al. 2020; Lacoursière‐Roussel et al. 2016; Zhang et al. 2022). The very high eDNA signal of *O. similis* could be due to several different reasons. There will be temporal variation in water column biodiversity due to seasonal reproductive events, which has the potential to skew abundances detected (Cleary et al. 2025). Water stratification, water currents, and the depth preferences of the different copepod species may also be a contributing factor (Harrison et al. 2019; Jeunen et al. 2020; Lacoursière‐Roussel et al. 2018; Xiong et al. 2025). Given such a busy shipping route, there are no baseline studies on how water turbulence from ships affects mixing of the water column and subsequent eDNA signals. Furthermore, there is still huge uncertainty about how long eDNA signals remain in the water after shedding and whether this timescale differs with species (Harrison et al. 2019; Jeunen et al. 2020). Finally, whilst primer bias is a known problem in molecular biodiversity surveys, this is ascribed to the presence or absence of a species which is expected in a particular dataset, and to date, the efficiency of detection using universal primers, and therefore abundance level, is rarely evaluated (Elbrecht and Leese 2015; Zhan et al. 2014). Sagittoidea, a marine predatory worm class, often contributes the most to zooplankton biomass after copepods, in concurrence with Sagittoidea being the most abundant non‐copepod Animalia ASV detected with COI (Hopcroft et al. 2005; Kosobokova and Hirche 2009). However, Sagittoidea was only the fourth most abundant Animalia class detected with 18S, proceeded by the gastropod *L. retroversa* and the hydrozoan *Aglantha digitale* , reflecting different primer biases. Difficulties amplifying members of Gastropoda and Cnidaria with COI have previously been reported (Christianson et al. 2022; Günther et al. 2018). Diatoms (Class Bacillariophyceae) were more effectively captured with the 18S primer set, largely represented by the centric diatom genus *Chaetoceros*. *Chaetoceros* has previously been reported as the dominant species during diatom blooms when sufficient nitrate and light are available (Ardyna and Arrigo 2020; Ardyna et al. 2011). Similarly, the prasinophyte *M. pusilla* , the second most abundant ASV detected with COI, is dominant in Arctic phytoplankton communities, and anticipated to benefit from longer ice‐free seasons and warmer, more‐stratified surface waters (Ardyna and Arrigo 2020; Hoppe et al. 2018). Our survey also detected numerous potentially harmful or toxic genera such as the diatom *Pseudo‐nitzschia*, prymnesiophyte *Chrysochromulina*, and dinoflagellates *Alexandrium*, *Dinophysis*, *Karenia*, *Karlodium*, and *Procentrum* (Poulin et al. 2011).

Baffin Bay had the highest alpha diversity across the survey area, detected with both primer sets. This is unsurprising as Baffin Bay is often considered one of the most productive regions within Arctic Canada, due to increased vertical mixing and high phytoplankton biomass (Ardyna et al. 2011). Productivity has been decreasing in Baffin Bay in recent decades, with the dominant centric diatom *Chaeteros* spp. decreasing as smaller protists, such as dinoflagellates, and picoeukaryotes, such as *Micromonas,* become more abundant (Ardyna and Arrigo 2020; Blais et al. 2017). This trend is reflected in our results, with the Dinophyceae class being the most abundantly detected ASV across both primer sets and consistently dominating read proportions in Baffin Bay and across the study area. Conversely, samples collected in Pond Inlet, within Eclipse Sound, had the lowest alpha diversity, especially with 18S, and the community composition is distinct from other samples collected in the east of the study region, which all clustered together. These differences were not significant, likely due to the small number of samples collected all at the same location off the coast of Pond Inlet. This area is encompassed within the Tallurutiup Imanga National Marine Conservation Area, acknowledged for its ecological and cultural significance, and has experienced the highest increases in vessel traffic in Arctic Canada (Halliday et al. 2022). Therefore, further work is warranted to investigate whether lower species diversity and different community composition could be linked to increased shipping traffic, including the presence of ships causing physical or noise disturbance and pollution in the waters. Knowledge holders in Pond Inlet have previously reported decreased numbers of marine mammals during the shipping season as a result of increased shipping (van Luijk et al. 2022). There also appears to be a distinction between community composition in James Ross/Rae Straits and Queen Maud Gulf, compared to the rest of the surveyed areas. This area is generally shallower, with higher sea surface temperature and salinity, likely contributing to differences observed in the community (Figures S1–S3). There was a peak in abundance for the copepod *P. newmani* in Rae Strait and Queen Maud Gulf with COI, and for *M. longa* in James Ross Strait with 18S. *Pseudocalanus* spp. are often more prevalent in shallower areas, so this is unsurprising, but *M. longa* usually makes up a larger proportion of copepod biomass in deeper waters (> 200 m) (Ashjian et al. 2003; Darnis et al. 2022). The peak in abundance of *T. nordenskioeldii* aligns with previous findings that *Thalassiosira* spp. are more abundant in the central archipelago compared to Lancaster Sound and Baffin Bay (Crawford et al. 2018).

### 4.2 Amphibalanus improvisus

To the best of our knowledge, this study reports the most northerly detections (68°–74° N) of the highly invasive *A. improvisus* to date (Meng et al. 2024) (Figure S4). *A. improvisus* is one of the most successful invaders worldwide, spreading from the western Atlantic across temperate and tropical regions (Carlton et al. 2011). Early successful invasions in Europe and along the western United States coast date back to the 1850s, largely associated with hull fouling (Carlton et al. 2011; Meng et al. 2024; Stasolla et al. 2021). *A. improvisus* 's success as an invasive species has been attributed to its broad environmental tolerance, being both euryhaline and eurythermal, ability to self‐fertilize, high reproductive and establishment rates, and an opportunistic diet (Nasrolahi, Pansch, Havenhand, and Wahl 2012; Pansch et al. 2013; Stasolla et al. 2021; Sundell et al. 2019). *A. improvisus* has previously been identified as high risk for establishing successfully in Arctic Canada (Goldsmit et al. 2018), with live specimens found on hulls in the Port of Churchill and surviving transportation through Arctic Canada waters (Chan et al. 2015, 2016). We are confident that the detections in this study represent *A. improvisus* in the environment as opposed to internal or external fouling of the survey vessel, as the barnacle was detected in samples from both the survey vessel's sea chest and Niskin bottles and not consistently detected across all samples. We cannot rule out that the detections represent fouling on other vessels, but limited shipping traffic was detected within 10 km of the samples being collected. Generally, eDNA is estimated to persist in the marine water column between 2 and 48 h, although future research on persistence rates and potential movement in the cold Arctic waters would be beneficial, as the cold water could enable eDNA to persist for longer, up to a period of 2 weeks (Collins et al. 2018; McCartin et al. 2022). The detections of *A. improvisus* show a sensible pattern in abundance, with the highest abundance in the east and declining along the transect, potentially indicating that *A. improvisus* has invaded from the east and started to spread. Pond Inlet, the most easterly community within the study area, has experienced the highest levels of vessel traffic increases of any Canadian Arctic community, providing a vector for *A. improvisus* to enter Arctic Canada (Dawson et al. 2018). Further, Pond Inlet is often the first stop for international ships arriving in Arctic Canada, as it is the most efficient location for passport control. Therefore, this particular port is highly interconnected within the global shipping network (Saebi et al. 2020). Furthermore, with ships having to moor up or remain in the vicinity for passport processing of passengers and crew, there is a greater chance of depositing *A. improvisus* propagules from the hulls of these ships into the water column in significant numbers than would be expected with transient passages (Carlton et al. 2011; Meng et al. 2024) (Figure S4).

We cannot determine with eDNA whether detections of *A. improvisus* represent sessile juveniles/adults or transient non‐settling larvae. *A. improvisus* larvae have been detected year‐round in the water column in the Western Baltic and Kiel Fjord where sea temperatures reach as low as 2°C, suggesting larvae would be able to persist in parts of Arctic Canada (Javidpour et al. 2009; Thomsen et al. 2010). However, environmental requirements for metamorphosis from the final cyprid larvae stage to an attached juvenile have only been tested down to 12°C (Nasrolahi, Pansch, Lenz, and Wahl 2012). High intracellular concentrations of the amino acid proline and aquaglyceroporins contained within their genome, which can act as freeze tolerant or antifreeze agents respectively, suggest *A. improvisus* has the capability to adapt to colder conditions outside of its normal range (Sundell et al. 2019). Recent advances in environmental RNA (eRNA) methodology may be useful for describing population demographics in the future, with *A. improvisus* being an ideal candidate as it undergoes large developmental changes and metamorphosis, increasing the likelihood of detecting differences in gene expression between life stages (Kalke et al. 2020; Parsley and Goldberg 2024). Understanding whether these detections represent larvae or sessile life stages will be important to assess the magnitude of impact that *A. improvisus* may have. It is highly likely that the detection of *A. improvisus* detailed here is due to pelagic larvae in the water column. Previous eDNA studies aimed at comparing water column biodiversity with benthic sediments have shown little connectivity between the two (Antich et al. 2021; Brandt et al. 2021). A more recent study by Cleary et al. (2025) specifically aimed at determining if eDNA signals in the water column could be linked to the composition of benthic communities again showed largely distinct representation of the two communities. However, close correlations between the two communities were observed in this survey when sampling water close to the seabed, when the weight on the Niskin bottle accidentally hit the seabed prior to water sampling. The resultant sediment plume was captured within the water samples and in these cases, there was strong representation of the soft sediment communities in the ‘water column’ sample. Such detections are unlikely with organisms restricted to hard rock substrata. Therefore, the presence of potentially invasive hard rock colonizing species in the water column should be regarded as an early warning signal to trigger a more detailed monitoring alert with local communities. Elsewhere, *A. improvisus* has outcompeted native barnacle species to become the dominant barnacle, so could impact native populations of barnacles or shellfish in Arctic Canada (Goldsmit et al. 2023; Mirzajani et al. 2016). Further, this occurrence of *A. improvisus* signifies that invasive species can be transferred to Arctic Canada, with the potential for increased invasive species arrival events likely in the future.

### 4.3 Future Recommendations for eDNA as a Monitoring Tool in the Arctic

In this study, we demonstrate the utility of using commercial ships as a platform for conducting eDNA biodiversity surveys in Arctic Canada to assess ongoing impacts of increased human activities and climate change. Previous eDNA studies in Arctic Canada have either been constrained to easily accessible, small spatial areas, such as ports, or relied on research vessels to cover larger, more remote spatial areas (Jacquemot et al. 2024; Lacoursière‐Roussel et al. 2018; Leduc et al. 2019; Sevellec et al. 2021). The distance traveled by cruise ships in Arctic Canada (NORDREG Zone) has more than doubled since 1990, especially in the Northwest Passage, and these ships travel both along main shipping corridors and into unique and often biologically, ecologically, and culturally significant areas (Dawson et al. 2021). Further, Goldsmit et al. (2019) highlight through an ecological risk assessment that domestic ship discharge also poses a high risk of dispersal. Therefore, national and international cruise ships represent a useful and feasible alternative to more expensive and sometimes less mobile research vessels for undertaking large‐scale systematic surveys, especially for sampling methods such as seawater collection for eDNA analyses, which do not rely on complex scientific equipment. Cruise ships potentially offer numerous advantages over research vessels including: (1) low carbon sample collection as sampling is carried out on an existing vessel route without the requirement for an additional research vessel, (2) cost‐effective, assuming cruise ship operators are willing to collaborate with scientists and host them for minimal costs in contrast to commissioning a research expedition and (3) repeatable, as cruise ships reliably follow the same routes annually, permitting long‐term monitoring to effectively capture any changes in community composition over time such as the arrival of new species (Boyse et al. 2023; Valsecchi et al. 2021).

eDNA surveys paired with citizen science initiatives and collaboration with local Canadian Arctic communities could further increase the spatiotemporal scales of biodiversity monitoring in the Arctic and identify the first signs of potentially invasive species, triggering an early warning alert for more detailed investigation. For example, successful citizen science‐based eDNA studies elsewhere have allowed nationwide surveys to be carried out simultaneously across the country through engagement with large volunteer networks (Agersnap et al. 2022). Biodiversity monitoring in Arctic Canada is currently largely limited to a small temporal window when the sea ice is absent allowing vessels to enter the area. Therefore, the involvement of local communities in eDNA surveys could be particularly important for increasing the temporal scale of surveys in Arctic Canada and increasing our understanding of biological community composition under the ice. Not only do citizen science initiatives increase the spatiotemporal scope of eDNA sample collection, but they can also be utilised to confirm the physical presence of an invasive species previously detected with eDNA in the water column (Encarnação et al. 2021). For example, collaboration with Canadian Arctic communities where the barnacle was detected (i.e., Pond Inlet and Gjoa Haven), or through citizen science schemes with tourists on cruise ships at other locations, could potentially confirm the presence of sessile adult bay barnacles in coastal intertidal areas.

## 5 Conclusion

In this study, we have proven that eDNA metabarcoding represents a highly sensitive monitoring tool for invasive species detections in the Arctic, through our eDNA survey being the first to detect the highly invasive bay barnacle ( *A. improvisus* ) in Arctic Canada. Simultaneously, eDNA metabarcoding can also provide critical baseline data on current community composition, making it an extremely cost‐effective multi‐aim monitoring tool. We recommend increased collaboration with citizen science initiatives and local indigenous communities to further increase both the spatiotemporal collection of eDNA samples alongside physical monitoring for colonization of invasive species (highlighted by eDNA alerts) and to ensure long‐term monitoring of a healthy marine environment. Increased monitoring efforts are essential to ensure that the arrival of any new non‐indigenous species is captured early to limit any potential negative impacts to Arctic ecosystems and Inuit communities.

## Author Contributions

**Elizabeth Boyse:** data curation, formal analysis, methodology, visualization, writing – original draft, writing – review and editing. **Melody S. Clark:** conceptualization, funding acquisition, supervision, writing – review and editing. **Ian M. Carr:** formal analysis, resources, software. **Alison J. Cook:** software, visualization. **Philippe Archambault:** conceptualization, funding acquisition, writing – review and editing. **Jean E. Holloway:** project administration, writing – review and editing. **Zhewen Luo:** formal analysis, resources. **Michael Milton:** conceptualization, resources. **Mathieu Roy:** data curation. **Jackie Dawson:** conceptualization, funding acquisition, project administration, writing – review and editing. **Victoria L. Peck:** conceptualization, data curation, funding acquisition, project administration, supervision, writing – review and editing.

## Conflicts of Interest

The authors declare no conflicts of interest.

## Supporting information


**Data S1:** gcb70452‐sup‐0001‐DataS1.xlsx.

**Data S2:** gcb70452‐sup‐0002‐DataS2.pdf.

## Data Availability

The data that support the findings of this study are openly available in the NCBI Bioproject repository under accession number PRJNA1204286.
