# Supplementary material for: Expanding Monitoring Capacity for Potential Invasive Species in Arctic Canada With Environmental DNA Metabarcoding
Source: Glob Chang Biol. 2025 Sep 8;31(9):e70452. doi: 10.1111/gcb.70452 (PMC12415680; doi:10.1111/gcb.70452)
Supplement: Supplementary file 2 — Data S2: gcb70452‐sup‐0002‐DataS2.pdf. [file GCB-31-e70452-s001.pdf]

Supplementary Information

Expanding monitoring capacity for potential invasive species in Arctic Canada with environmental DNA metabarcoding

**Supplementary Table 1**	Metadata for environmental DNA sampling locations.
**Supplementary Table 2**	Mean/SE of reads lost across the DADA2 workflow.
**Supplementary Figure 1**	Map showing bathymetry in the study area.
**Supplementary Figure 2**	Map showing mean daily SST during the sampling period.
**Supplementary Figure 3**	Map showing mean daily salinity during the sampling period.
**Supplementary Figure 4**	Global distribution of *Amphibalanus improvisus.*

Supplementary Table 1. Metadata for each environmental DNA sample collected along the Northwest Passage Sea Route in Arctic Canada.

Name	Date	Time	Type	Location	Marine Region	Latitude	Longitude	Bathymetry (m)	Sea Surface Temperature (°C)	Salinity (‰)
**U1.1**	23/08/2023	09:36	Underway	Mid Baffin Bay	Baffin Bay	73.158	-65.745111	-2363	4.86	30.64
**F1.1**	24/08/2023	11:30	Fixed	Pond Inlet	Eclipse Sound	72.7	-77.988111	-23	NA	30.76
**U1.2**	25/08/2023	08:54	Underway	Dundas Harbour	Lancaster Sound	74.5335	-83.1757	-58	NA	NA
**F1.2**	25/08/2023	16:30	Fixed	Croker Bay	Lancaster Sound	74.819389	-83.175694	-129	NA	NA
**U1.3**	25/08/2023	16:30	Underway	Croker Bay	Lancaster Sound	74.819389	-83.175694	-129	NA	NA
**U1.4**	26/08/2023	10:11	Underway	Radstock Bay	Lancaster Sound	74.775306	-90.9005	-74	NA	NA
**F1.3**	26/08/2023	16:00	Fixed	Beechey Island	Lancaster Sound	74.718889	-91.802611	-16	NA	NA
**U1.5**	26/08/2023	17:00	Underway	Beechey Island	Lancaster Sound	74.718889	-91.802611	-16	NA	NA
**F1.4**	27/08/2023	08:40	Fixed	Port Leopold	Prince Regent Inlet	73.862	-90.301389	-24	3.17	NA
**U1.6**	27/08/2023	12:23	Underway	Port Leopold	Prince Regent Inlet	73.862	-90.301389	-24	3.17	NA
**U1.7**	27/08/2023	15:33	Underway	East Somerset Island	Prince Regent Inlet	73.377306	-90.804111	-287	2.76	NA
**U1.8**	28/08/2023	08:20	Underway	Bellot Strait	Bellot Strait	72.0015	-94.737	-85	NA	NA
**F1.5**	28/08/2023	16:24	Fixed	Conningham Bay	Franklin Strait	71.798389	-96.815944	-25	NA	NA
**U1.9**	28/08/2023	16:24	Underway	Conningham Bay	Franklin Strait	71.798389	-96.815944	-25	NA	NA
**U1.10**	29/08/2023	10:54	Underway	East King William Island	James Ross/Rae Straits	69.9025	-96.535194	-32	1.93	26.34

**U1.11**	29/08/2023	19:41	Underway	East Matty Island	James Ross/Rae Straits	69.359306	-94.038889	-4	NA	NA
**F1.6**	30/08/2023	09:30	Fixed	Gjoa Haven	James Ross/Rae Straits	68.617806	-95.887111	-8	NA	NA
**U1.12**	31/08/2023	17:11	Underway	Cambridge Bay	Queen Maud Gulf	69.111611	-105.0585	-25	NA	NA
**U2.1**	01/09/2023	08:01	Underway	South King William Island	Queen Maud Gulf	68.647389	-98.249806	-43	4.94	NA
**U2.2**	02/09/2023	11:47	Underway	West Mainland before Bellot Strait	Franklin Strait	70.703806	-97.340694	-79	1.87	26.56
**U2.3**	03/09/2023	11:37	Underway	Roy Fitz Fjord opening	Franklin Strait	72.121694	-95.178694	-165	NA	NA
**U2.4**	03/09/2023	14:35	Underway	East Bellot Strait	Prince Regent Inlet	71.999194	-94.290111	-11	1.96	NA
**U2.5**	04/09/2023	22:04	Underway	Somerset Island Devon Island Crossing	Lancaster Sound	74.2055	-89.762611	-208	2.31	29.1
**U2.6**	05/09/2023	21:43	Underway	Radstock Bay Entrance	Lancaster Sound	74.417194	-88.415194	-319	2.7	29.85
**U2.7**	06/09/2023	21:31	Underway	Devon Island Bylot Island Crossing	Lancaster Sound	74.165306	-80.879889	-799	2.94	26.8
**U2.8**	07/09/2023	07:05	Underway	East Bylot Island	Baffin Bay	72.920889	-75.905889	-123	3.09	30.85
**U2.9**	08/09/2023	07:50	Underway	Baffin Bay	Baffin Bay	72.206194	-70.598694	-1579	2.51	31.35

Supplementary Table 2. Mean and Standard Error (SE) of reads lost across the DADA2 workflow.

Primer set	Sample type	Mean reads with primers removed	SE	Mean reads filtered	SE	Mean reads denoised	SE	Mean reads merged	SE	Mean reads with chimeras removed	SE
**18S**	Field	70,104	1468	64,236	1391	63,788	1383	61,866	1349	60,620	1312
**18S**	Blank	3452	427	2908	387	2814	383	2684	364	2672	363
**COI**	Field	107,102	5792	92,904	5317	92,370	5301	89,720	5243	87,824	5212
**COI**	Blank	136	22	82	19	58	19	49	15	49	15

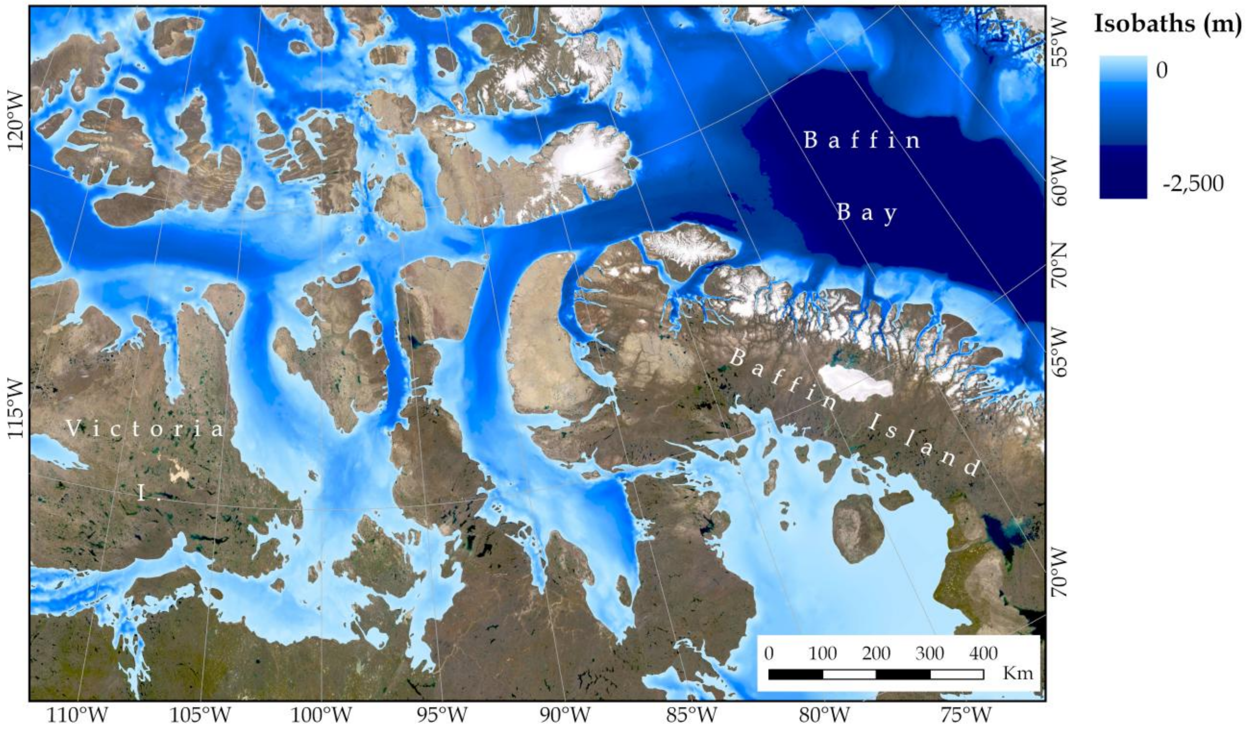

Supplementary Figure 1. Map displaying the General Bathymetric Chart of the Oceans (GEBCO) 2024 bathymetry grid (0.004x0.004°) for the study area in the Canadian Arctic.

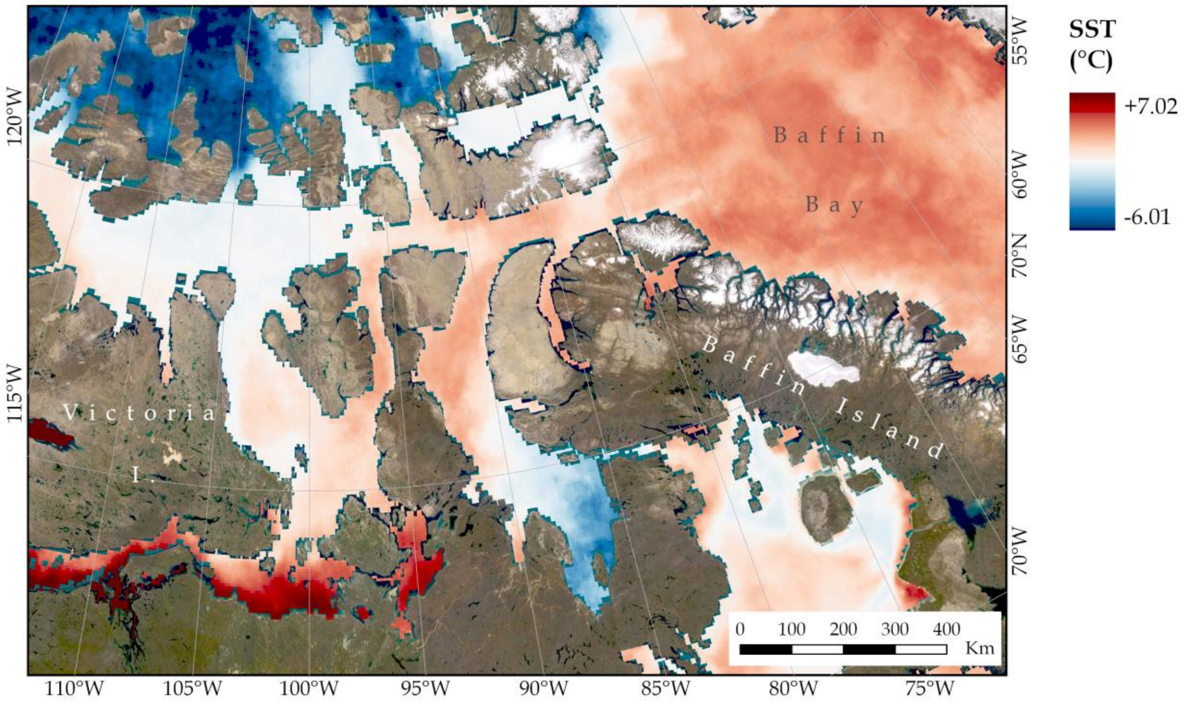

Supplementary Figure 2. Map displaying mean daily sea surface temperature (SST) at 0.05x0.05° resolution during the sampling period (23/08/2023 – 08/09/2023; https://doi.org/10.48670/moi-00130).

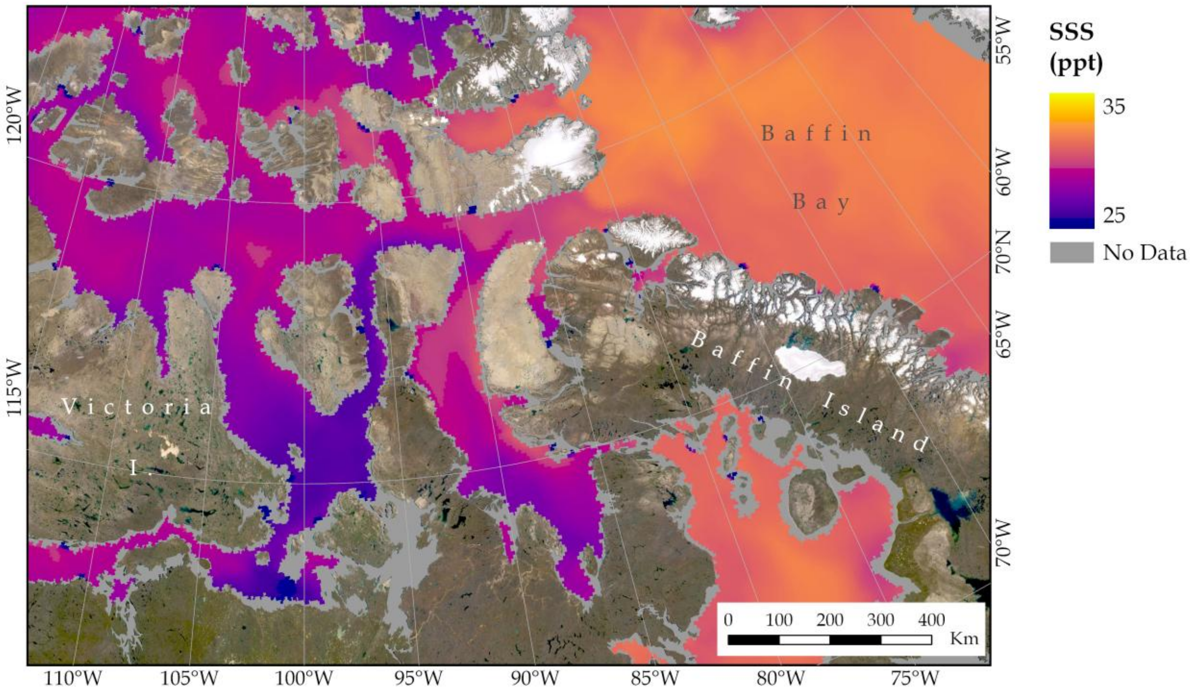

Supplementary Figure 3. Map displaying daily mean sea surface salinity (SSS) at 0.08x0.0625° resolution during the sampling period (23/08/2023-08/09/2023; https://doi.org/10.48670/moi-00001).

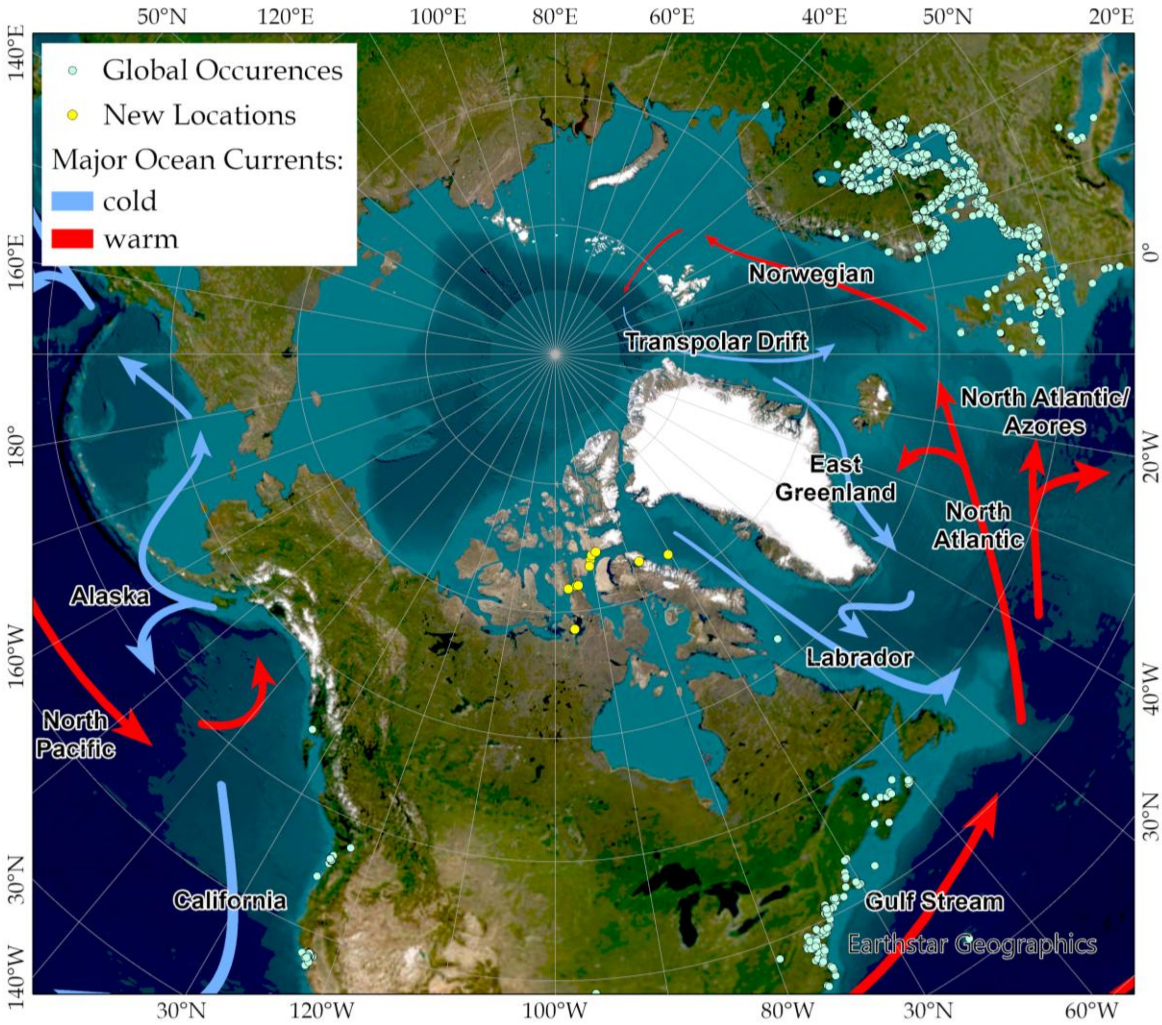

Supplementary Figure 4. Global occurrences of *Amphibalanus improvisus* from GBIF (https://doi.org/10.15468/dl.krgqsz) highlighted in blue, and new occurrences detected in the high Canadian Arctic with eDNA highlighted in yellow.
